# High Accuracy Decoding of Movement Target Direction in Non-Human Primates Based on Common Spatial Patterns of Local Field Potentials

**DOI:** 10.1371/journal.pone.0014384

**Published:** 2010-12-21

**Authors:** Nuri F. Ince, Rahul Gupta, Sami Arica, Ahmed H. Tewfik, James Ashe, Giuseppe Pellizzer

**Affiliations:** 1 Brain Sciences Center, VA Health Care System, Minneapolis, Minnesota, United States of America; 2 Department of Neuroscience, University of Minnesota, Minneapolis, Minnesota, United States of America; 3 Department of Electrical and Electronics Engineering, Cukurova University, Adana, Turkey; 4 Department of Electrical and Computer Engineering, University of Minnesota, Minneapolis, Minnesota, United States of America; The University of Western Ontario, Canada

## Abstract

**Background:**

The current development of brain-machine interface technology is limited, among other factors, by concerns about the long-term stability of single- and multi-unit neural signals. In addition, the understanding of the relation between potentially more stable neural signals, such as local field potentials, and motor behavior is still in its early stages.

**Methodology/Principal Findings:**

We tested the hypothesis that spatial correlation patterns of neural data can be used to decode movement target direction. In particular, we examined local field potentials (LFP), which are thought to be more stable over time than single unit activity (SUA). Using LFP recordings from chronically implanted electrodes in the dorsal premotor and primary motor cortex of non-human primates trained to make arm movements in different directions, we made the following observations: (i) it is possible to decode movement target direction with high fidelity from the spatial correlation patterns of neural activity in both primary motor (M1) and dorsal premotor cortex (PMd); (ii) the decoding accuracy of LFP was similar to the decoding accuracy obtained with the set of SUA recorded simultaneously; (iii) directional information varied with the LFP frequency sub-band, being greater in low (0.3–4 Hz) and high (48–200 Hz) frequency bands than in intermediate bands; (iv) the amount of directional information was similar in M1 and PMd; (v) reliable decoding was achieved well in advance of movement onset; and (vi) LFP were relatively stable over a period of one week.

**Conclusions/Significance:**

The results demonstrate that the spatial correlation patterns of LFP signals can be used to decode movement target direction. This finding suggests that parameters of movement, such as target direction, have a stable spatial distribution within primary motor and dorsal premotor cortex, which may be used for brain-machine interfaces.

## Introduction

Brain-machine interface (BMI) technology, in which brain signals are used to control external prostheses, has been applied successfully in non-human primates [1]-[6] as well as in human subjects [7]. The basic principle of invasive BMI applications is to relate neural activity, such as extra-cellular potentials from cortical neurons, to movement parameters such as direction [8], velocity [9], and position [1], and use this relation to control an external device. Currently, one of the major limitations that militate against the widespread use of BMI technology in human subjects is the lack of long-term stability in recordings of extra-cellular potentials from single cortical neurons. In this study, we examined the decoding power of local field potentials (LFP), which may also be recorded from indwelling electrodes. Since LFP represent the sum of synaptic dendritic potentials within a volume of cortex, these signals are thought to have greater stability in time compared to that of single neurons, making them potentially more suitable for BMI applications [10]. However, understanding the relation between LFP and motor behavior is still in its early stages. In this perspective, initial LFP decoding studies were not especially encouraging [11], [12], however more recent publications have shown that it was possible to decode information about motor responses from LFP in premotor [13], motor [14], [15] and posterior parietal cortex [16]. In addition, it was found that direction-related information could be extracted from both low and high frequency sub-bands of the LFP signal [13], [15], [17], and that direction information may even be extracted during an instructed-delay period [13]. However, despite the progress made to date on using LFP to extract information about response direction, the decoding power obtained using simultaneously recorded LFP, which is what would be required for effective BMI applications, has been relatively modest (∼0.5 with 8 LFP channels recorded simultaneously). Good decoding power (∼0.8) has been achieved only when data from 48 channels recorded in separate sessions were combined as if they had been recorded simultaneously [14], [15]. However, this practice is likely to overestimate the actual decoding performance of simultaneously recorded LFP channels.

In the current study, we had four major objectives: (i) to examine directional information in LFP signals both during an instructed-delay period before movement and during movement itself using chronically implanted multi-electrode arrays, (ii) to compare the strength of directional information from LFP in primary motor and dorsal premotor cortex, (iii) to compare the accuracy of directional information extracted from LFP with that extracted from SUA, and (iv) to investigate the stability of direction decoding across recording sessions. One of the innovative features of our study was to discriminate target directions by applying spatial filters on LFP. To our knowledge, this is the first study that used this approach.

## Materials and Methods

### Subjects and task

Two male rhesus monkey (Macaca mulatta), H464 and H564, were subjects in this study. Care and treatment of the animals conformed to the U.S. Public Health Service Policy on Humane Care and Use of Laboratory Animals (Public Law 99–158) and to the Guide for the Care and Use of Laboratory Animals (National Academy Press, 1996). Precautions were taken to insure the welfare of the non-human primates. They were housed in conditions which took account of their social needs and they participated in the institutional primate environment enrichment plan. No procedure that might cause discomfort or pain was undertaken without adequate analgesia or anesthesia as outlined in details below. The experimental protocol was approved by the Institutional Animal Care and Use Committee of the Minneapolis Veterans Affairs Health Care System.

The monkeys were trained in an instructed-delay center-out reaching task. They performed two-dimensional horizontal reaching movements in which they used a manipulandum to control the movements of a cursor on a visual display from a central location to one of eight peripheral targets equally spaced around a circle of ∼9 cm radius. To begin a trial, the subject placed the cursor inside a circular window (radius ∼1 cm) at the center of the display and held it for a control period of 800 ms. Then, a peripheral circular target was displayed pseudo-randomly at one of the eight locations and remained visible for 500–700 ms serving as a cue for the subject. An instructed-delay period of 800–1000 ms followed the offset of the cue, after which the target re-appeared at the same location as the cue, thus serving as a ‘GO’ signal. Successful movements were completed within 1000 ms and finally the subject had to hold the cursor for 800 ms within the target circle (radius ∼1 cm) to obtain a juice reward.

### Surgical procedures

All surgical procedures were performed under sterile conditions and under general anesthesia. The two animals underwent surgery to place head posts for head stabilization during neural recordings, and to implant two chronic recording arrays. The intracortical electrode arrays were placed subdurally in the arm area of M1 and PMd of the cerebral hemisphere contralateral to the hand performing the task. Preoperative preparation included the intramuscular injection of atropine (0.05 mg/kg) to manage bradychardia, and ketamine (10 mg/kg) to induce anesthesia. Intraoperative anesthesia was maintained via endotracheal administration of isoflurane (1–2%). A catheter was placed in the saphenous vein and 0.9% saline was administered for fluid management. A heating pad was used to support body temperature during surgery. Rectal temperature, heart rate, breathing rate and oxygen saturation were monitored continuously during surgery. Postoperative care included the intramuscular injection of dexamethasone (0.5 mg/kg) to reduce inflammation and enrofloxacin (Baytril 3.75 mg/kg) as antibiotic. In addition, the analgesic buprenorphine (Buprenex 0.05 mg/kg) was given intramuscularly twice a day for three days.

### Data Acquisition

We analyzed the data from three recording sessions in each subject; there was a gap of one week between sessions 1 and 2 and one day between sessions 2 and 3.

For H464, the number of trials was 520, 263, and 326 for sessions 1, 2 and 3, respectively. For H564, the number of trials was 199, 206, and 103 for sessions 1, 2 and 3, respectively. So, a total of 1109 and 508 trials were available for subjects H464 and H564, respectively. LFP signals were recorded using two 10×10 Utah microelectrode arrays (Blackrock Microsystems, Salt Lake City UT); these arrays were 4 mm ×4 mm in size, with an inter-electrode spacing of 400 µm and an electrode length of 1.5 mm. There were 96 active electrodes in each array from which we sampled 64 because we lacked a sufficient number of channel amplifiers for the full set of electrodes. In both monkeys, one array was implanted in M1 and one in PMd. LFP data were filtered between 0.3 Hz and 500 Hz and sampled at 1 kHz. Extracellular potentials were recorded simultaneously from both arrays during the performance of the task, filtered between 500 Hz and 7.5 kHz and sampled at 30 kHz. Only spike waveforms greater than a threshold determined for each recording session were stored for further processing.

### Signal Processing

Using visual inspection, we eliminated LFP channels containing artifacts, such as power line noise, from the analysis. The number of PMd channels available for analysis was 25 and 57 for H464 and H564, respectively; whereas the number of M1 channels available for analysis was 36 and 59, respectively. These channels were low-pass filtered with a 220 Hz cut-off frequency and down-sampled to 500 Hz for further analysis. As an initial step, we implemented a time-frequency analysis to identify sub-bands that were modulated during the task. For this purpose, the time-frequency surface was normalized relative to the average baseline power level during the session [18]. This analysis was implemented separately for channels in M1 and PMd. On these bases, the LFP signal was sub-band filtered into five different frequency bands in which we observed systematic changes during the task: 0.3–4 Hz, 4–10 Hz, 14–22 Hz, 22–30 Hz and 48–200 Hz. However, despite the high-pass filtering of 0.3 Hz during recording, we noticed DC artifacts in several trials; therefore the overall trial mean was subtracted from the data of each trial before sub-band filtering. The sub-band filtering was performed using linear phase finite impulse response (FIR) filters designed with a Blackman window function [19]. The filters were applied in a forward direction only. The phase delay was eliminated by shifting the signals backwards by *N*/2 samples, where *N* is the filter order. The order of the filter for each sub-band was adjusted such that signal attenuation at the cut-off frequencies was -6 dB. Following the sub-band filtering, the amplitude of the 0.3–4 Hz sub-band and the envelope of the signal of the higher frequency bands (from 4–10 Hz to 48–200 Hz), computed using Hilbert transform, were used for further processing [19]. The Hilbert transform is a useful tool for the analysis of the oscillatory components of time-varying signals. It is used to form a complex analytic signal composed of the real narrow band time-series and the imaginary Hilbert transform of that time-series. The magnitude of the analytic signal corresponds to the envelope of the time-series. In theory this procedure creates less distortion in the estimation of the envelope than using a half-wave or full-wave rectification. The time-varying envelopes of sub-band data were low-pass filtered using a FIR filter with a cut-off frequency of 30 Hz to prevent aliasing during the down-sampling operation. The signal in each sub-band was down-sampled to 100 Hz and processed for feature extraction. For analysis of the SUA, spike sorting was performed offline using the Offline Spike Sorter (Plexon, Inc., Dallas TX), primarily through manual isolation of spike clusters in two or three dimensional feature spaces.

### Feature Extraction and Classification

The decoding algorithm used in this study did not use directly the amplitude of LFP in different sub-bands, but used the correlation of this amplitude across electrodes. In other words, the algorithm extracted information regarding target direction from the spatial patterns of the LFP signals. We used this feature as the basis for our direction decoding strategy because we observed systematic changes in the correlation between LFP channels across target direction (see below). Consequently, we extracted features in each sub-band using a method that exploited the difference in correlation patterns between channels for different classes of target directions. For this purpose, we used the Common Spatial Patterns (CSP) algorithm [20]. The CSP method finds a set of projections of neural data such that the amount of variance obtained by linear combination of input channels is maximized for one class while it is minimized for another. The spatial projections *X*_CSP_ were computed as follows:
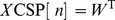
(1)


where *X* ∈ R^C^ represents the multi-channel sub-band data, *n* represents the time sample, C is the number of channels, whereas the columns of *W* ∈ R^CxC^ are the spatial filters. The projections *X*_CSP_[*n*] correspond to a weighted linear combination of input channels using the spatial filters *W*. The spatial filters *W* were found by a generalized eigenvalue decomposition method:
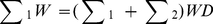
(2)


where ∑_1_ and ∑_2_ ∈ R^CxC^ are the spatial covariances estimated from the recording channels for two different classes of target direction, and *D* is the diagonal matrix of eigenvalues of ∑_1_. Once the spatial projections were computed, their log-transformed variance were calculated and served as feature vector for decoding. The log-transformation was used to normalize the skewed distribution of variance.

The CSP algorithm has been widely used as a feature extraction tool in multichannel neural activity processing for binary classification paradigms [21]. However, since we are tackling a multi-class problem (i.e., discrimination of 8 directions), we used a redundant classification strategy based on the fusion of the error correcting output codes (ECOC) method [22] with the CSP algorithm. In this scheme, we constructed several CSP filters and related linear classifiers to discriminate target direction within every possible pair of targets (28 contrasts: 0° vs. 45°, 0° vs. 90°, 0° vs. 135°, …, 270° vs. 315°), as well as to discriminate groups of 2–4 contiguous target directions from the opposite group of 2–4 contiguous targets (12 contrasts: {0°,45°} vs. {180°,225°}, {45°,90°} vs. {225°,270°}, …, {45°,90°,135°,180°} vs. {225°,270°,315°,0°}). In other words, we created a redundant classification strategy in which every direction was tested against every other direction and each group of two to four neighboring directions was tested against the corresponding diametrically opposite group. The overall system had a total of *L* = 40 binary classifiers (i.e., 28+12 contrasts). For each contrast, six features (i.e., the log-transformed variance of the projected data) were computed using the three largest and three smallest eigenvectors from *W* to construct a low dimensional feature representation. These features were submitted to a Fisher linear discriminant (FLD) classifier.

**Figure 1:**
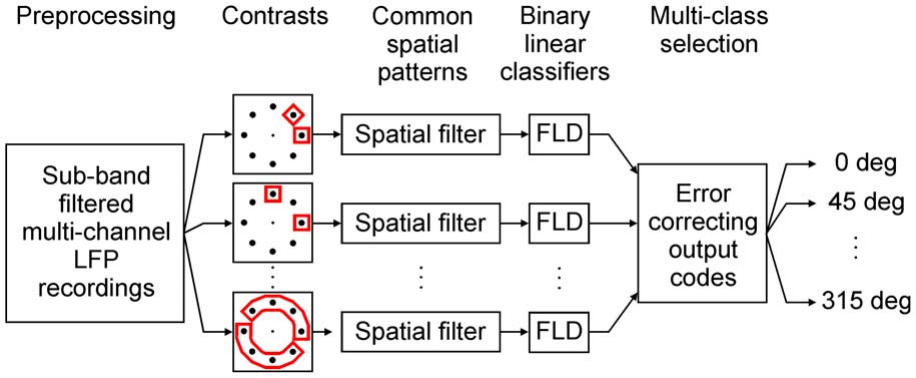
Schematic of the decoding algorithm. Sub-band filtered multichannel LFP were processed using spatial filters to decode movement target direction. Common spatial patterns (CSP) filters were constructed to contrast binary classes of target directions, as well as of groups of directions. The main features of the projected data from each spatial filter were fed to their related Fisher linear discriminant (FLD) classifier. Finally, the outputs from all FLDs were processed using the error correcting output codes (ECOC) for the multi-class decision.

The classifiers output of the redundant structure was then post-processed by ECOC to obtain a final decision. This last step was accomplished by multiplying the vector representing the classifiers output with the ECOC decoding matrix *M* of *K*x*L* with entries *m* ***_i,j_*** ∈ {−1, 0, 1} where *L* is the number of binary classifiers and *K* is the number of classes (i.e., 8 target directions). The index corresponding to the minimum value of the ECOC output was selected as the predicted direction of the test data. A schematic diagram of the decoding algorithm is given in Fig. 1. This algorithm has been described previously [23].

We computed the time-course of the directional decoding performance of the LFP signal aligned to two different behavioral events, cue onset and movement onset. For cue aligned data, we used a 500 ms window with an initial position 500 ms before cue onset and shifted it 13 times in 100 ms increments ending at 1200 ms after cue onset. For movement aligned data, we used a 1000 ms time window with an initial position 1400 ms before movement onset and shifted the window with 100 ms increments ending at 1000 ms after movement onset. With SUA data, we performed two types of analyses: one using the same CSP+ECOC algorithm as described above, and one using the regularized linear discriminant analysis (rLDA), which was shown to provide better results than other methods, such as the population vector and multivariate Gaussian modeling approaches [14]. In the first case, spike counts were computed in 2 ms windows resulting in a sampling frequency of 500 Hz like the LFP data. Then each time-series of spiking activity was sub-band filtered and processed for feature extraction and classification as described above. In the second case, the rLDA was performed on the spike counts computed in 200 ms windows, which was shifted along the time axis in 100 ms shifts.

### Statistical Analyses

We evaluated the decoding performance of the algorithm at each time-shift using a 10×10-fold cross-validation method. The decoding performance values reported correspond to the average over folds and repetitions of the cross-validation. Cross-validation provides a relatively unbiased estimate of the generalization capacity of the algorithm by separating the trials used for training from those used for testing. In the main analysis, the data of all three sessions were combined. However, we did also a number of analyses were data from one or two sessions were used for training, whereas data from the other session(s) were used for testing. The decoding performance was evaluated in terms of decoding power (DP) and circular correlation (ρT). DP is the ratio of correctly classified trials relative to all trials, and is the most commonly used measure of classification accuracy. However, DP does not take the spatial properties of direction into account. For example, DP does not differentiate between the cases where the system selects a direction close to the correct one or distant from it. In addition, DP does not take into account the circularity of direction (i.e., 0 = 360 deg). For these reasons, we also evaluated the quality of decoding using the circular correlation coefficient ρT [24]. We compared the decoding performance between sub-bands and between cortical areas using a corrected *t*-test [25], which has been shown to have appropriate Type I and Type II errors in the context of cross-validation procedures [26]. All p-values less than 0.05 were considered significant.

## Results

**Figure 2:**
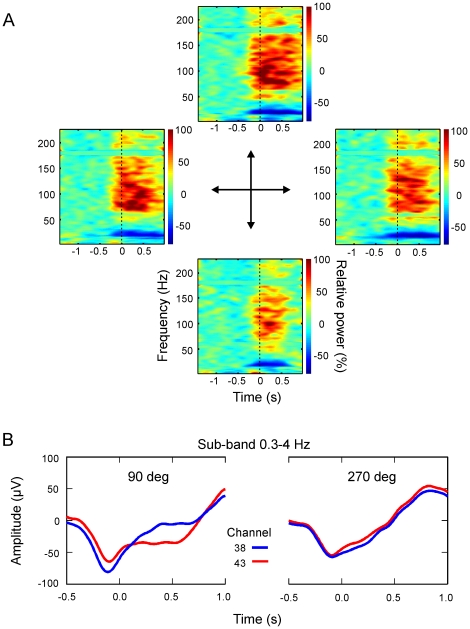
Modulation of LFP across target direction. (A) Time-frequency maps of LFP signals from one channel in M1 for 4 target directions (out of 8) during the instructed-delay center-out task. The data were aligned to the onset of movement (t = 0 s). The color map represents the change in power relative to baseline. The central arrows indicate the target direction associated with the different maps. The most reactive frequency bands were selected from these time-frequency maps for decomposing the LFP data into sub-bands. (B) Average LFP data in the 0.3–4 Hz band of two representative channels for the 90 deg and 270 deg target directions. The data were aligned to the onset of movement (t = 0 s). Note the difference in correlation between channels for these two directions. This characteristic was used for target direction decoding on a single trial basis.

**Figure 3:**
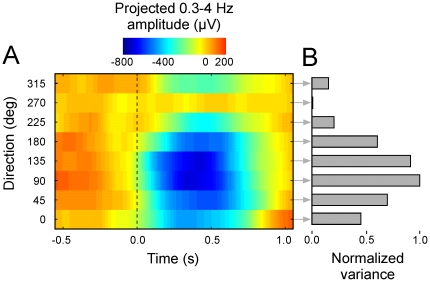
Projected data. (A) Projections of 0.3–4 Hz LFP data from all channels using a spatial filter tuned to maximize the variance for the 90 deg direction and minimize the variance for the 270 deg direction. The projections were ordered along the ordinate according to target direction. The vertical dashed line indicates movement onset. (B) Variance of the spatial projection for each target direction in Fig. 3A. The variance was normalized to the maximum value. Note that the variance was maximum for 90 deg and minimum for 270 deg by design of the spatial filter, and that it varied progressively across direction. This characteristic indicates that LFP correlation structure across channels changed progressively with direction.

We recorded neural activity in each of M1 and PMd during the performance in the instructed-delay task across three recording sessions. There were systematic changes in the time-frequency power of the LFP signal across target direction. Examples of time-frequency maps for LFP data from one channel are illustrated in Fig. 2A. For the analysis, we separated the signal into five different frequency bands: 0.3–4 Hz, 4–10 Hz, 14–22 Hz, 22–30 Hz and 48–200 Hz. Furthermore, since different channels had different LFP patterns across direction, the correlation between signals from different channels changed with direction, as well. An example of average movement-onset LFP data in the 0.3–4 Hz band of two representative channels for two target directions, 90 deg and 270 deg, is shown in Fig. 2B. The figure illustrates the difference in correlation between signals from the two channels for these diametrically opposite directions. This characteristic was the basis of the single trial direction decoding strategy used in the current study. The CSP algorithm exploits the differences in correlation across all channels for different directions to discriminate between them. Fig. 3A shows the average projection of LFP data in the 0.3–4 Hz sub-band using a spatial filter that maximized the variance for the 90 deg direction data and minimized the variance for the 270 deg direction data. The normalized variance of the projected data is plotted across direction in Fig. 3B. It shows that the variance of the projected data had a broad profile, which is evidence that the patterns of correlation changed progressively across target direction.

### Directional information across frequency bands and cortical areas

**Figure 4:**
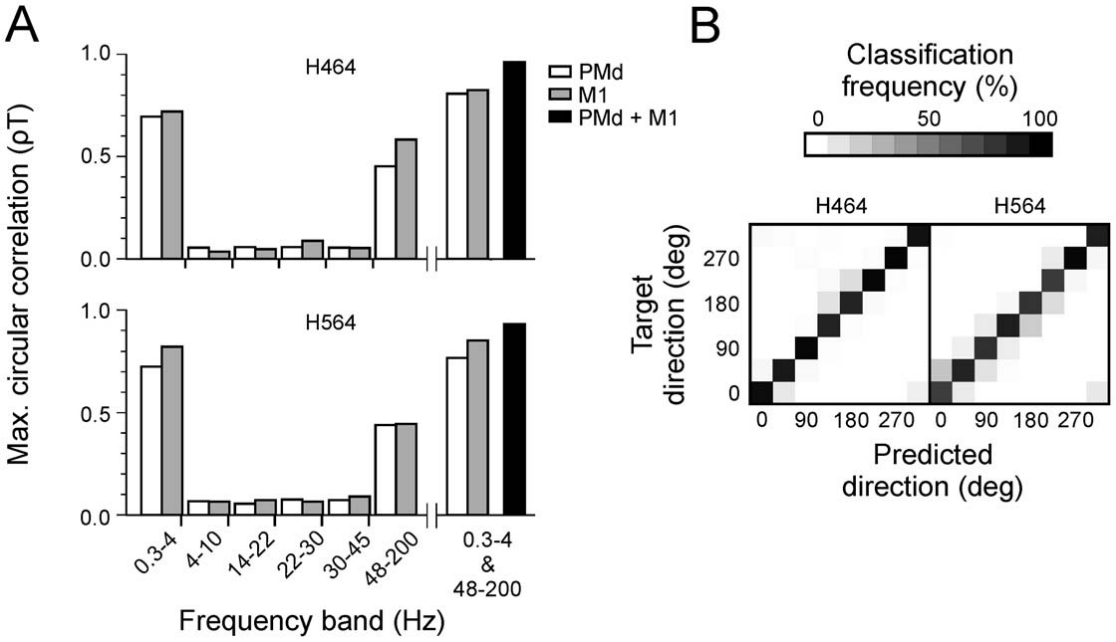
Decoding accuracy and confusion matrices. (A) Maximum circular correlation between predicted and actual target direction for each sub-band, brain area and subject. The results correspond to the cross-validated maximum correlation obtained during a trial. (B) Confusion matrices of decoding results using the 0.3–4 Hz and 48–200 Hz frequency bands combination from M1 and PMd data for each subject. A perfect prediction would result in all classifications being along the diagonal. Note that misclassifications occurred typically in neighboring directions evidencing that LFP for neighboring directions had similar correlation structures.

We calculated the maximum directional information that could be extracted from the LFP signal during single trials from each sub-band and each cortical area. The results in terms of circular correlation between predicted and actual target direction are illustrated in Fig. 4A. These results were obtained using data merged from the three recording sessions and by executing a 10×10-fold cross-validation procedure. There was a clear differentiation in information content across frequency bands in both subjects and for both cortical areas. Only two of the frequency sub-bands provided any notable direction information: the delta (0.3–4 Hz) and the upper gamma (48–200 Hz) bands. For both sub-bands and for both monkeys and brain areas, the maximum decoding accuracy was observed when the data came from a 1 s data window that started 0–0.3 s before movement onset. More details about the time-course of decoding accuracy are provided below. Although both of these sub-bands yielded circular correlations of at least 0.4, the circular correlations for the delta sub-band were significantly higher (all pair-wise *t*-tests with p<0.0001 for each combination of monkey and brain area), reaching values greater than or equal to 0.7. As might be expected, we obtained even better decoding accuracy (ρT >0.8) when we combined the LFP activity from the 0.3–4 Hz and 48–200 Hz frequency bands by concatenating the features originating from these sub-bands. The latter results are illustrated by the three rightmost bars in each sub-plot of Fig. 4A. Finally, we combined the features of the delta and gamma bands across both cortical areas, which improved the maximum circular correlation between decoded and actual direction further to 0.96 and 0.93 for H464 and H564, respectively (c.f. black bars in Fig. 4A). In terms of decoding power, the combination of features of the two sub-bands and two cortical areas led to 93% and 84% accuracy for subjects H464 and H564, respectively. In order to inspect the misclassifications produced by the system, we constructed confusion matrices of predicted versus actual target direction associated with the maximum decoding performance. Fig. 4B displays the results, which show that misclassified directions were typically adjacent to the actual direction. In other words, even when the classification failed, the predicted target direction was close to the actual direction. This is consistent with the idea that the patterns of correlation across channels changed progressively across direction.

### Time-course of directional information

**Figure 5:**
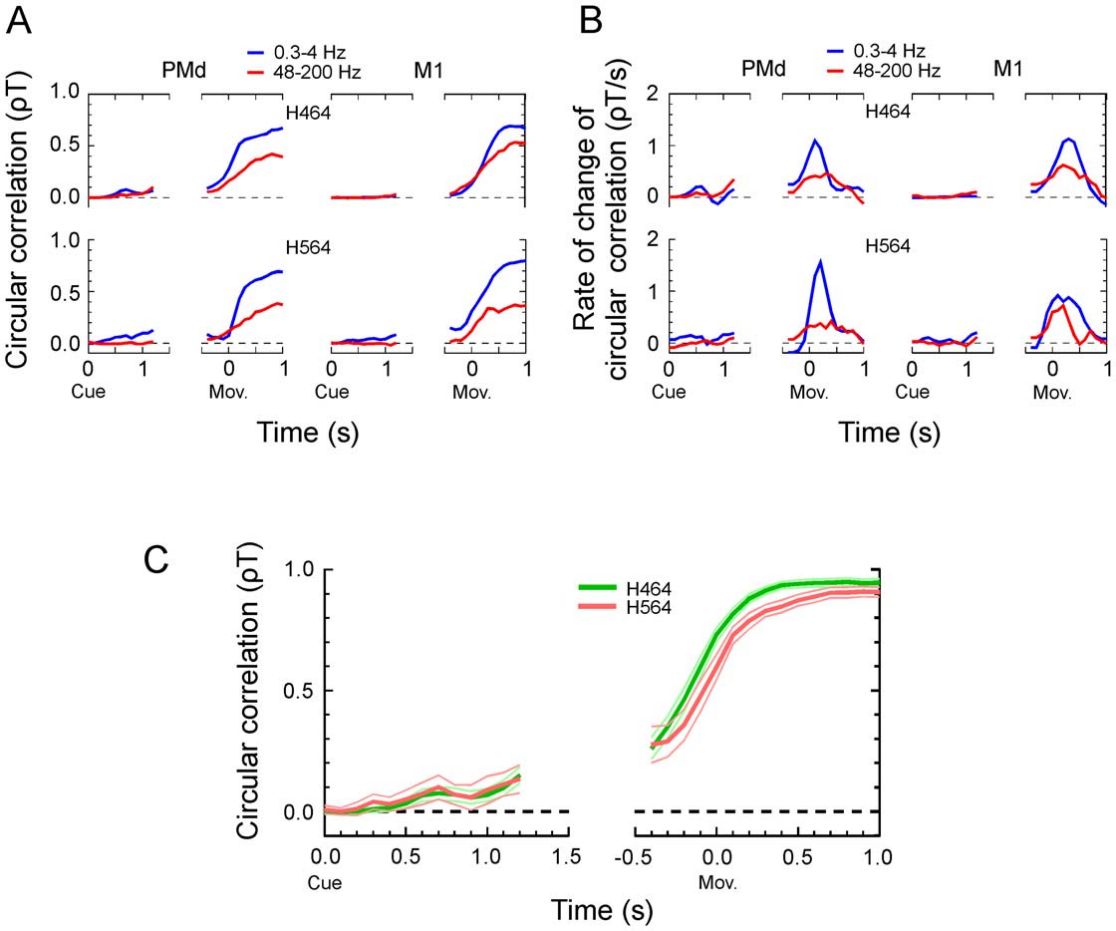
Temporal evolution of decoding accuracy. (A) Time-varying circular correlation between predicted and actual target direction for the two most informative sub-bands, and for each brain area and subject. The results at each time point were obtained using data from a time-window (500 ms for the cue and 1000 ms for movement onset aligned data, respectively) that preceded that time point. (B) Time-varying rate of change of circular correlation between predicted and actual target direction. Same conventions as in A. (C) Time-varying circular correlation between predicted and actual target direction using combined features obtained with the delta and upper gamma sub-bands from PMd and M1 for each subject. The thinner lines indicate the 95% confidence interval of the mean computed on the basis of the corrected *t*-test [25]; [ 26].

Having identified the two sub-bands that carried directional information, we examined the time-course of decoding accuracy during the task for these sub-bands, their combination, and for each subject and cortical area. The results are illustrated in Fig. 5A. First, the figure shows that there was low-level but consistent directional information during the instructed-delay-period in both monkeys. Since the correlation can vary from −1 to +1, even low level correlation but consistent over time can be useful to predict the upcoming direction of response. To determine whether the correlation was consistently positive during the delay period, we analyzed the sum of the correlation values across time-shifts during the delay period and found that it was significantly different from zero for the 0.3–4 Hz sub-band of PMd data (*t*-tests with p<0.0001 for both monkeys). This was also true, but to a lesser extent for the same sub-band in M1 (p = 0.040 for H464 and p<0.0001 for H564). In contrast, for the 48–200 Hz sub-band only the data from PMd in one subject (H464) were significantly different from zero (*t*-test with p<0.0001; all other *t*-tests with p>0.05). These results indicate that low level but consistent target direction information can be extracted from LFP during the instructed-delay period. Second, with movement-onset aligned data, we found that the accuracy of directional decoding increased progressively and reached its highest levels when the data window overlapped the onset of movement. In addition, the dynamics of the time-course of decoding accuracy was different between sub-bands and between brain areas. The difference can be better appreciated in Fig. 5B where the rate of change of circular correlation was plotted for each animal, sub-band and brain area. The peak of the rate of change of correlation was significantly higher for the 0.3–4 Hz sub-band than for the 48–200 Hz sub-band in both brain areas and for both monkeys (all *t*-tests with p≤0.011). In addition, the time following the peak at which the rate of change fell below 50% of peak amplitude occurred significantly earlier in PMd than in M1 (*t*-tests; p = 0.007 for H464, and p = 0.028 for H564). In summary, we would like to stress two general observations about these dynamics. First, the increase in decoding accuracy with time (using movement-onset aligned data) was step-like for the 0.3–4 Hz band, whereas it was ramp-like for the 48–200 Hz band in both brain areas. Second, the peak of the rate of change in decoding accuracy occurred earlier in PMd than in M1, that is, the decoding accuracy leveled-off earlier in PMd than in M1.

Next, we examined the time-course of decoding by combining data from the two sub-bands and the two brain areas. The results of this analysis are plotted in Fig. 5C. As expected from the results described above, we found that during the delay period, the correlation between predicted and actual target direction, though relatively low, was consistently positive. The correlation summed across time-shifts during this period was significantly different from zero (*t*-tests with p<0.0001 for both monkeys). In addition, the correlation during the peri-movement period reached very high levels (ρT >0.90) for both animals.

### Decoding stability

An important point of the current study was to test the stability of the LFP-based decoding across recording sessions. The fact that a high level of decoding accuracy was obtained when the data were combined over a period of eight days (Fig. 4A) supports the assumption that LFP data had consistent spatio-temporal characteristics across that period of time. As an additional test of signal stability, we performed decoding analyses using data from one or two sessions for training and used the data from the other session(s) for testing. The results are presented in Table 1. As it could be expected, there was a noticeable decrease in decoding accuracy when the training of the algorithm was based on data from a single session. However, there was no systematic effect on the results whether the data for training were from the first, second or last session. In addition, when the data of two sessions were used for training, then there was little or no decrease in decoding accuracy compared to the case where all sessions were combined. These results indicate that the characteristics of LFP were relatively stable across the sessions, and that using data across multiple sessions provides a more robust extraction of the consistent patterns.

**Table 1:** Decoding accuracy (DP: decoding power; ρT: circular correlation) across recording sessions.

	Training session(s)→Testing sessions(s)											
	1→2,3		2→1,3		3→1,2		1,2→3		1,3→2		2,3→1	
Subject	DP	ρT	DP	ρT	DP	ρT	DP	ρT	DP	ρT	DP	ρT
H464	0.36	0.27	0.71	0.76	0.64	0.68	0.87	0.91	0.92	0.95	0.60	0.62
H564	0.68	0.80	0.56	0.67	0.46	0.49	0.85	0.91	0.83	0.90	0.65	0.73

Data from the training session(s) were used to train the decoder, whereas data from the test session(s) were used to examine the decoding accuracy. There was a gap of 7 days between session 1 and 2, whereas session 2 and 3 took place in consecutive days.

### Directional information in LFP and SUA

In order to compare the amount of direction-related information in LFP and SUA, we decoded movement target direction for each type of signal using the CSP+ECOC algorithm. These analyses were performed for the two sessions in which both types of data were available (sessions 2 and 3). In addition, to prevent potential ambiguity about the identity of SUA across sessions, we performed the SUA analysis in each session separately and compared it to the LFP data in the same session. The number of isolated units for subjects H464 and H564 was 27 and 56 in session 2 and 24 and 58 in session 3, respectively. The best decoding power with SUA was obtained using the 0.3–4 Hz sub-band, which results are indicated in Table 2. The results show that decoding accuracy using LFP was generally slightly better than using SUA for both subjects in both sessions. These results can be explained in part by the larger number of LFP channels available than SUA. The effect of number of channels on decoding accuracy is examined in the next section. In addition, there was a clear drop in decoding accuracy for subject H564 in the last session due to the smaller number of trials available in that session for training the classification system. A large number of training trials is needed to accurately estimate the spatial covariance matrices and robustly extract the related filters. The effect of number of trials available for training the decoder is examined more in details further below. Finally, we investigated the accuracy of directional decoding with SUA using rLDA [14] to compare it with the accuracy obtained with the CSP+ECOC algorithm. The decoding accuracy in terms of circular correlation with SUA and using rLDA was 0.82 and 0.92 for monkey H464 in sessions 2 and 3, respectively; and 0.65 and 0.59 for monkey H564 in sessions 2 an 3, respectively. Consequently, the CSP+ECOC algorithm provided generally a better decoding of SUA compared to that of rLDA.

**Table 2:** Decoding accuracy (ρT) with SUA and LFP in single sessions.

	Session									
	2					3				
Subject	SUA	LFP	#SUA	#LFP	#Trials	SUA	LFP	#SUA	#LFP	#Trials
H464	0.92	0.98	27	61	263	0.91	0.98	24	61	326
H564	0.80	0.88	56	116	206	0.70	0.63	58	116	103

In all cases the decoding was performed using the CSP+ECOC algorithm.

### Directional information as a function of number of channels

**Figure 6:**
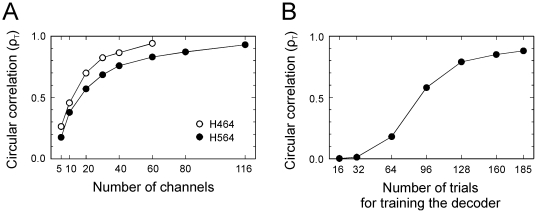
Decoding accuracy varies with number of channels and trials. (A) Circular correlation between predicted and actual target direction computed as a function of number of channels used for decoding. (B) Circular correlation between predicted and actual target direction computed as a function of number of trials used for training the decoding algorithm (data of H564 in session 2).

Decoding accuracy is dependent, among other things, on the number of data channels available. Therefore, in order to assess the decoding accuracy as a function of number of channels, we selected subsets of different sizes from the set of channels available for each monkey (i.e., 61 channels for H464 and 116 channels for H564). For each subset, the channels were randomly selected with replacement and a 10×10-fold cross-validation procedure was performed. The decoding accuracy in terms of circular correlation is shown in Fig. 6A. We observed a similar relation between number of channels and decoding accuracy in both subjects. This relation was characterized by a rapid increase in decoding accuracy when the number of channels increased from 5 to about 30. However, after 30 channels the classification performance increased much more slowly.

### Directional information as a function of number of trials

Finally, the more trials are available, the better the statistics estimates used for decoding. For this reason, we assessed the decoding accuracy as a function of number of trials available for training the decoder. In this analysis, we selected the data of subject H564 in session 2. Subsets with different number of trials were formed and the decoding accuracy was estimated using the 10×10-fold cross-validation procedure. In order to prevent bias towards a particular direction in these subsets, the number of trials was constrained to be the same across direction. The decoding accuracy in terms of circular correlation is shown in Fig. 6B. As expected, the decoding accuracy increased with number of trials used for training the decoder. It increased mainly for subsets up to 128 trials (i.e., 16 trials per direction) and leveled off thereafter.

### Benchmark for real-time processing

We tested whether our approach could be practical for real-time applications by implementing the algorithm described above using Matlab (MathWorks, Natick MA) on a 2.2 GHz CPU personal computer. The benchmark was executed using 128 channels of simulated LFP data sampled at 1 kHz. In the preprocessing step, the streaming input LFP data were filtered in the delta (0.3–4 Hz) and gamma (48–200 Hz) frequency bands. The envelope of the gamma component was computed using the Hilbert transform as described in the methods section. In the following step, the preprocessed data in both bands were low-pass filtered and down-sampled to 100 samples per second and processed by the CSP+ECOC algorithm for feature extraction and classification. Since the final sampling frequency was 100 Hz, each decision update should be given in less than 10 ms to provide a real-time feedback. We observed that with our current hardware setup, the preprocessing step took 5.6 ms, whereas the CSP+ECOC post-processing step was accomplished in 2.6 ms resulting in a total response time of about 7.3 ms. This result shows that the algorithm can be executed on traditional computer architectures in real-time.

For applications where the decoding is executed on the entire reach, as we did in this study, a frequent update rate is not necessary. On the other hand for trajectory decoding applications a fast update rate is indispensible. In this latter case, the time window analyzed has to be short as well to extract local features of the time-varying movement parameters. However, a short time window might produce a poor estimation of the CSP features. In this case, recursive methods, which can update adaptively the CSP features on a sample basis, might be needed [27].

## Discussion

We believe that the results we presented make a number of important points in regard to the decoding of directional information from LFP signals. First, it is possible to obtain high fidelity decoding of movement target direction over time from the patterns of chronically recorded multi-channel LFP; second, the direction of the intended movement can be extracted during a delay period before movement begins; third, the dynamics of direction decoding are different across sub-bands within the LFP signal; fourth, signals from the primary motor cortex and dorsal premotor cortex have similar decoding power though the dynamics are different; and finally the LFP signal is relatively stable over time.

### Decoding power and the spatial properties of the LFP signal

We have shown that it is possible to decode movement target direction with high accuracy by exploiting the spatial patterns of LFP recorded from M1 and PMd. As mentioned above, others have already used LFP signals to decode the direction of movement [14]–[17]. However, high levels of decoding power were achieved only when data channels were combined across recording sessions [14], [15], which is not practical for real-time BMI applications. We believe that the high decoding ability we demonstrated was related to the analytical approach which was based on the assumption that the LFP signal sampled from regularly placed electrodes on the cortical surface has a consistent spatial organization. The spatial organization of the LFP signal was supported by other aspects of our results in addition to the high decoding power. For example, we observed that when misclassifications occurred these were generally in favor of neighboring directions (Fig. 4B), which is understandable if neighboring directions are associated with similar structures in the LFP spatial patterns. In addition, in other work, we have shown that grouping LFP data from neighboring directions to train spatial filters and classifiers provided better classification results than when directions were treated as independent [23].

Since the observation that many neurons in the motor cortex are tuned to the direction of movement in space and that individual neurons have different preferred directions of movement [8], there has been much speculation as to whether the direction properties of single neurons in the motor cortex are organized in a regular topographic pattern similar to the columnar organization of orientation selectivity and ocular dominance in visual cortex [28]. There is now evidence from single cells recording that their preferred movement direction may be organized in a columnar network [29]–[31]. Although our study does not address this question directly, it provides support for the concept of spatial organization of movement-related neuronal properties. Notwithstanding, it is not yet clear how the directional properties of single neurons and LFP signals might relate, which is a question beyond the scope of the current experiment.

### Stability of LFP in time

We were able to address two aspects of the relation between the LFP signals and time. The first was whether the LFP signal yielded stable decoding of the parameter of interest over the three recording sessions which spanned a period of eight days. We obtained very high decoding accuracy (Figs. 4A and 5A) when the data of all the sessions were combined, which suggests that there was a consistent pattern across channels during these sessions that could be used to decode movement target direction. The second aspect of stability over time was whether patterns of neural activity in one or two experimental sessions could be used to train the algorithm and predict target direction using the neural patterns from one or more other sessions. To this end, we performed decoding analyses in which we trained the decoding algorithm using data from one or more experimental sessions and then decoded data from one or more other sessions (Table 1). The two main aspects of the results were that which session data were used for training the decoder did not have a clear systematic effect on decoding accuracy (i.e., the worst performance was found when session 1 was used for training with monkey H464, whereas it was when session 3 was used for training with monkey H564; on the other hand, the best performance was obtained when sessions 1 and 3 were used for training with monkey H464 and sessions 1 and 2 with monkey H564); and that using the data of two or three sessions for training increased noticeably the decoding accuracy over using the data of a single session. These results support the hypothesis that the characteristics of LFP were relatively stable across the sessions, and that a robust extraction of spatial patterns may require more than one session. Overall, these results suggest that although there are common spatial patterns that appear to be stable over time, there is in addition a non-stationary process in the LFP signal that needs to be further characterized and may pose a challenge to decoding algorithms.

### SUA and LFP decoding

Decoding of movement parameters based using single-unit activity is currently regarded as the gold-standard for BMI applications. The data from single units recording have the advantage of fine spatio-temporal precision, high information content and a vast literature on their relation to various aspects of behavior. However, a major difficulty in the use of SUA is the difficulty in isolating the same neurons over time [7], [10]. In terms of signal, it was shown that good neuronal signals can be recorded from chronic arrays for, at least, 1.5 years [32], [33]. However, an important question is whether the same neurons can be recorded over multiples sessions. Studies that have addressed this question by analyzing the stability of SUA over time have found that less than half of the originally isolated neurons were still available after 1–2 weeks [34], [35]. For these reasons, in some studies only a small subset of stable SUA was used for evaluating the decoding accuracy over a two week period [36], [37]. In other words, these studies discarded a large amount of recorded SUA, because they were not consistent across sessions. In contrast to SUA, LFP is considered to be a potentially more stable signal. In addition, it was shown that LFP can be used to decode movement parameters such as direction, velocity and position with accuracy similar to that of SUA [14]. The current study shows that movement target direction can be decoded with high accuracy using the spatial patterns of LFP. However, pairs of LFP channels tend to be more correlated than SUA or MUA channels [38] which means that increasing the number of LFP channels typically increases redundancy. This is consistent with the negatively accelerated gain in decoding accuracy with number of channels (Fig. 6A), that is, the slope of the relation between decoding accuracy and number of channels was steeper with fewer than 30 channels and shallower afterwards. These results underscore the difficulty with estimating decoding performance when channels recorded across different sessions are added together as if they were independent channels [14], [38]. This practice is likely to overestimate the actual decoding performance of simultaneously recorded LFP channels.

In addition, we show that, generally, better direction decoding was obtained with LFP than with SUA. This result may be accounted for in part by the fact that there were fewer SUA available than LFP channels. Indeed, when the number of SUA and LFP channels are similar, SUA yield better decoding results than LFP [38]. Our study supports these results to some extent. For example, when we consider a similar number of LFP channels (i.e., 30 and 60 for H464 and H564, respectively) and SUA (i.e., 27 and 56 for H464 and H564, respectively), then the decoding results were 0.82 and 0.83 with LFP and 0.92 and 0.75 with SUA for H464 and H564, respectively. In summary, with a comparable number of channels, SUA provided better decoding results than LFP for subject H464, whereas LFP provided better decoding than SUA for subject H564. In conclusion, the superiority of the LFP signal is predicated upon having an adequate amount of data; if the data are limited, as can be seen in one case in Table 2, then the performance of the SUA may be better. However, since LFP signals represent the summed synaptic activity over a volume of neural tissue, it is expected that typically more channels with stable LFP recordings be available than the number of isolated SUA.

### Decoding in M1 and PMd

The primary motor cortex is the most important brain area for the control of voluntary movements [39]. We have detailed information about the encoding of motor parameters in motor cortex and neural data from this structure have been the focus of the majority of decoding studies that are relevant for BMI applications [1], [3]–[7]. However, for practical reasons it would be unwise to focus exclusively on the primary motor cortex as the location from which signals might be decoded. The area of motor cortex that is readily accessible on the cortical surface is relatively small and there is a variety of diseases in which this structure may be damaged making it unusable. The premotor areas on both the lateral and medial surface of the frontal lobe contribute to the planning of movements, the integration of somatosensory and visual information essential for movement, and to the production of movement sequences [40]–[42]. In addition, elemental parameters of movement, particularly direction, are typically encoded in premotor areas, such as the PMd, well in advance of the movement itself and before the appearance of activity in M1 [43], [44]. The ability to decode in advance information about an upcoming movement is essential if BMI applications are to improve. In the current experiment, we were able to detect a small but significant signal related to the upcoming movement target direction during a delay period almost a second before the subject moved. In addition, we found that the dynamics of change in neural signal were different in PMd compared to M1 just before and during movement. Finally, the overall decoding accuracy though somewhat lower in PMd was still comparable to that of M1.

### Conclusions

The results demonstrate that the spatial patterns of LFP signals can be used to decode movement target direction. This finding suggests that parameters of movement, such as target direction, have a stable spatial pattern within primary motor and dorsal premotor cortex, which may be used for brain-machine interfaces.
